# Obesity affects pulmonary function in Japanese adult patients with asthma, but not those without asthma

**DOI:** 10.1038/s41598-022-20924-y

**Published:** 2022-09-30

**Authors:** Hiroki Tashiro, Koichiro Takahashi, Yuki Kurihara, Hironori Sadamatsu, Yuki Kuwahara, Ryo Tajiri, Shinya Kimura, Naoko Sueoka-Aragane

**Affiliations:** 1 Division of Hematology, Respiratory Medicine and Oncology, Department of Internal Medicine Faculty of Medicine, Saga University, 5-1-1 Nabeshima, Saga, Saga 849-8501 Japan; 2 Clinical Research Center, Saga University Hospital, Saga, Japan

**Keywords:** Respiration, Physiology, Diseases, Respiratory tract diseases

## Abstract

Obesity is associated with the severity of asthma, which is characterized by airway obstruction. Pulmonary function testing is one of the important examinations for evaluating airway obstruction. However, the impact of obesity on pulmonary function in patients with asthma is not fully understood. A total of 193 patients with asthma and 2159 patients without asthma who visited Saga University Hospital were investigated retrospectively. Obesity was defined as a body mass index (BMI) greater than 25 kg/m^2^. Pulmonary functions including forced vital capacity (FVC) and forced expiratory volume in 1 s (FEV_1_) were compared between patients with and without asthma, focusing especially on obesity. FVC percent predicted and FEV_1_ percent predicted were significantly lower in patients with asthma than in those without asthma (*p* = 0.03, < 0.01 respectively). In patients with asthma, FVC percent predicted and FEV_1_ percent predicted were significantly lower in patients with obesity than in those without obesity (all *p* < 0.01). In addition, BMI was negatively correlated with FEV_1_ (*r* =− 0.21, *p* = 0.003) and FVC (*r* = − 0.15, *p* = 0.04), along with the percent predicted. On multivariate analysis in patients with asthma, FVC (β [95% confidence interval] 0.12 [0.02–0.22], *p* = 0.02) and FEV_1_ (0.13 [0.05–0.22], *p* < 0.01) were still significantly different between patients with and without obesity. However, these obesity-associated differences were not observed in patients without asthma. Obesity reduces pulmonary function, including FVC and FEV_1_, in patients with asthma, but not in those without asthma.

## Introduction

Asthma is a common respiratory disease the pathophysiology of which involves airway inflammation and airway hyperresponsivity^1–3^, which induce respiratory symptoms such as shortness of breath, coughing, and wheezing due to narrowing of the airway^4,5^. To evaluate the disease control level precisely and objectively, pulmonary function testing has been widely recognized as a useful tool^6^.

Pulmonary function testing is one of the important examinations for patients with asthma, and forced expiratory volume in 1 s (FEV_1_) in particular is a parameter that reflects the status of disease control related to airway obstruction^7,8^. For example, corticosteroid therapy, which is a pivotal treatment for asthma, increases FEV_1_ dramatically^9^, and, in contrast, severe asthma patients showed decreased FEV_1_ even with intense treatment^10,11^. In addition, a previous report showed that a decreased FEV_1_ is associated with poor outcomes in patients with asthma^12^. These data indicate that exploration of factors related to reduced FEV_1_ is important for the management of asthma in daily clinical care.

Obesity and asthma are closely related, and obesity contributes to clinical outcomes of patients with asthma^13^. For example, obesity increases the incidence of asthma beyond age and race differences^14^. Obesity also affects the severity of asthma, and for severe asthma patients in the United States, the prevalence of obesity was 57.3% in adults, which is substantially higher than that in the general United States population^15^. In addition, the annual rate of exacerbation, which is one of the characteristics of asthma severity, is higher in asthma patients with obesity than in those without obesity in Japan^16,17^. As for the mechanisms, it is considered that obesity increases systemic and airway inflammation and induces resistance to corticosteroid therapy, which augments asthma pathophysiology^18–21^. In terms of pulmonary function, it can be reduced by the excess adipose tissue on the chest wall in patients with obesity compared to those without obesity^22,23^. However, the impact of obesity on pulmonary function in patients with asthma is not fully understood. The aim of the present study is to assess the impact of obesity on pulmonary function in patients with asthma.

## Results

### Characteristics of patients with and without asthma

**Figure 1:**
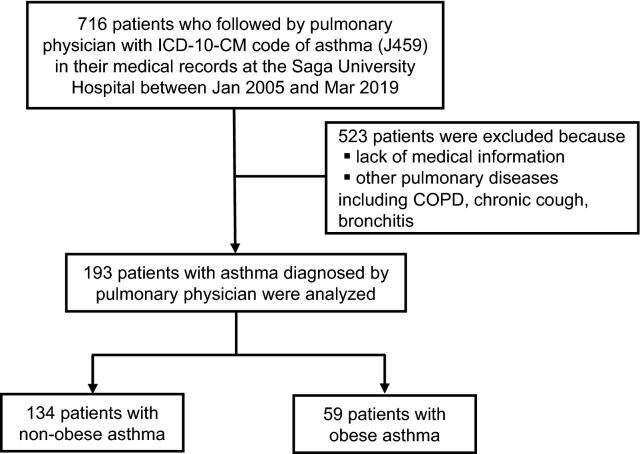
Study design: Patients with asthma. A total of 193 patients with asthma diagnosed by a pulmonary physician were identified, and 134 non-obese patients with asthma and 59 obese patients with asthma were analyzed.

**Figure 2:**
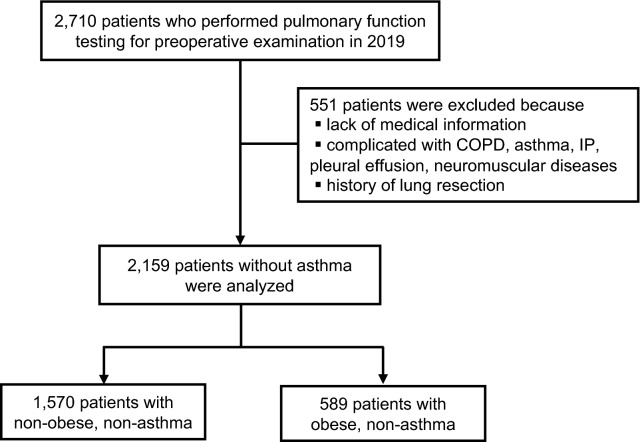
Study design: Patients without asthma. A total of 2159 patients without asthma were extracted, and 1570 non-obese patients without asthma and 589 obese patients without asthma were analyzed.

First, 193 patients with asthma and 2159 patients without asthma were analyzed (Figs. 1, 2). The body mass index (BMI) and height were not different between the groups. Patients with asthma were significantly younger than those without asthma (*p* < 0.01). There were more females and fewer with a smoking history in patients with asthma than in those without asthma (both *p* < 0.01). There were more never smokers in patients without asthma than in those with asthma (*p* = 0.01), but the rates of ex-smokers and current smokers were not different between the two groups. For comorbidities, the rates of hypertension, diabetes mellitus, and cardiovascular diseases were significantly lower in patients with asthma than in those without asthma (all *p* < 0.01), and the rate of hyperlipidemia was not different between the groups. On pulmonary function testing, FEV_1_ percent predicted and FVC percent predicted were significantly lower in patients with asthma than in those without asthma (*p* = 0.03, < 0.01 respectively) (Table 1). On univariate analysis, FEV_1_ (β [95% confidence interval] 0.11 [0.06–0.17], *p* < 0.01), but not FVC (0.04 [− 0.02–0.1], *p* = 0.24), was significantly different between the patients with and without asthma. On multivariate analysis adjusted by height, age, sex, and smoking history with respect to known cofounding factors for asthma incidence, pathophysiology, and pulmonary function, FVC (0.06 [0.03–0.1], *p* < 0.01) and FEV_1_ (0.17 [0.14–0.2], *p* < 0.01) were significantly different between patients with and without asthma. In particular, there was a corresponding reduction in FVC of 0.06 L and FEV_1_ of 0.17 L for patients with asthma compared to those without asthma (Table 2). Next, 189 patients from each group were extracted by the propensity score-matching (PSM) method to mitigate the risk of confounding due to differences between patients with and without asthma. Comorbidities such as hypertension, diabetes mellitus, hyperlipidemia, and cardiovascular diseases were not different, but FVC percent predicted, FEV_1_, and FEV_1_ percent predicted were still significantly different (*p* = 0.03, < 0.01, < 0.01 respectively) (Supplemental Table 1).

**Table 1:** Characteristics of patients with and without asthma.

	Patients without asthma	Patients with asthma	*p* value
*n*	2159	193	
Body mass index (kg/m^2^)	23.0 ± 4.1	23.0 ± 3.8	0.98
Height (cm)	159.6 ± 9.4	158.6 ± 9.1	0.23
Age	62.9 ± 17.0	53.2 ± 19.5	< 0.01
Sex (M/F)	1128/1031	69/124	< 0.01
Smoking history (pack-year)	14.5 ± 24.0	8.9 ± 18.5	< 0.01
**Smoking status**			
Never smoker	1257 (58.2%)	130 (67.4%)	0.01
Ex-smoker	549 (25.4%)	40 (20.7%)	0.14
Current smoker	353 (16.4%)	23 (11.9%)	0.1
**Comorbidities**			
Hypertension	895 (41.5%)	51 (26.4%)	< 0.01
Diabetes mellitus	406 (18.8%)	21 (10.9%)	< 0.01
Hyperlipidemia	446 (20.7%)	29 (15.0%)	0.05
Cardiovascular diseases	347 (16.1%)	16 (8.3%)	< 0.01
**Pulmonary function test**			
FVC, percent predicted (%)	96.5 ± 15.1	93.5 ± 17.1	0.03
FEV_1_, percent predicted (%)	95.9 ± 15.2	83.4 ± 20.9	< 0.01

FVC, forced vital capacity; FEV_1_, forced expiratory volume in 1 s.

**Table 2:** Univariate and multivariate analyses of FVC and FEV_1_ in patients with asthma versus patients without asthma.

Patients with asthma versus patients without asthma	*β*	95% CI	*p* value
**Univariate analysis**			
FVC (L)	0.04	− 0.02–0.1	0.24
FEV_1_ (L)	0.11	0.06–0.17	< 0.01
Multivariate analysis*			
FVC (L)	0.06	0.03–0.1	< 0.01
FEV_1_ (L)	0.17	0.14–0.2	< 0.01

FVC, forced vital capacity; FEV_1_, forced expiratory volume in 1 s; CI, confidence interval.

*FVC and FEV_1_ were individually adjusted by confounding factors including height, age, sex, and smoking history.

### Impact of obesity in patients with and without asthma

**Figure 3:**
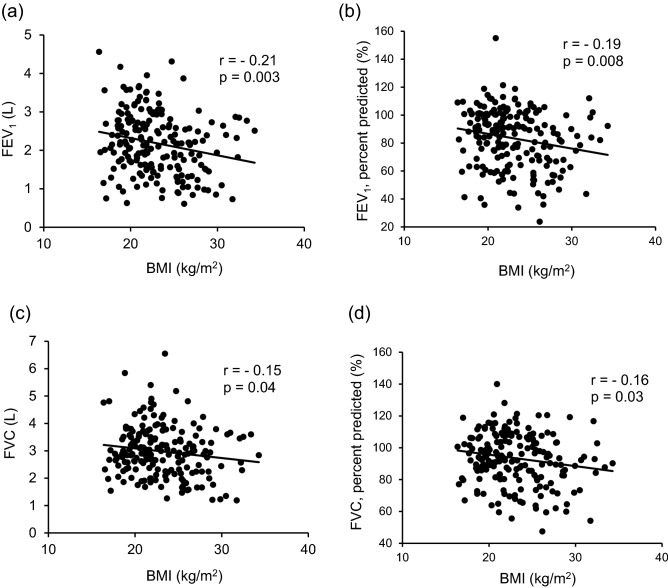
Correlations between forced expiratory volume in 1 s, forced vital capacity, and the body mass index in patients with asthma. (**a**) The forced expiratory volume in 1 s (*r* = − 0.21, *p* = 0.003) and (**b**) the forced expiratory volume in 1 s, percent predicted (*r* = − 0.19, *p* = 0.008) are significantly negatively correlated with the body mass index. (**c**) The forced viral capacity (*r* = − 0.15, *p* = 0.04) and (**d**) the forced vital capacity, percent predicted (*r* = − 0.16, *p* = 0.03) are correlated with the body mass index, though the correlation coefficient is low.

Of the patients without asthma, 1,570 were non-obese (average BMI 21.1 kg/m^2^), and 589 were obese (average BMI 28.2 kg/m^2^). Age, sex, smoking history, and smoking status were not different. The rates of hypertension, diabetes mellitus, hyperlipidemia, and cardiovascular diseases were significantly higher in obese patients without asthma than in non-obese patients without asthma (*p* < 0.01. < 0.01, < 0.01, = 0.02, respectively). Parameters of pulmonary function testing including FVC percent predicted and FEV_1_ percent predicted were not different (Table 3). Additionally, FVC and FEV_1_ were not different even after adjustment for confounding factors including age, sex, and smoking history on multivariate analysis (Tables 4). Of the patients with asthma, 134 were non-obese (average BMI 20.9 kg/m^2^), and 59 were obese (average BMI 27.7 kg/m^2^). Obese patients with asthma were significantly older than non-obese patients with asthma (*p* < 0.01), but sex, smoking history, and smoking status were not different between the two groups. The rates of hypertension, diabetes mellitus, and hyperlipidemia, but not of cardiovascular diseases, were significantly higher in obese patients with asthma than in non-obese patients with asthma (*p* < 0.01, < 0.01. < 0.01, 0.09, respectively). Parameters of pulmonary function testing including FVC percent predicted and FEV_1_ percent predicted were significantly lower in obese patients with asthma than in non-obese patients with asthma (all *p* < 0.01) (Table 3). In addition, BMI was negatively correlated with FEV_1_ (*r* = − 0.21, p = 0.003), FEV_1_, percent predicted (*r* = − 0.19, *p* = 0.008), FVC (*r* = − 0.15, *p* = 0.04), and FVC percent predicted (*r* = − 0.16, *p* = 0.03), even though the correlation coefficients were low (Fig. 3a–d). On univariate analysis, FVC (0.23 [0.09–0.36], *p* < 0.01) and FEV_1_ (0.26 [0.14–0.38], *p* < 0.01) were significantly different between patients with and without obesity. On multivariate analysis with adjustment for age, sex, smoking history, and ICS dose, FVC (0.12 [0.02–0.22], *p* = 0.02) and FEV_1_ (0.13 [0.05–0.22], *p* < 0.01) were still significantly different between patients with and without obesity. In particular, there was a corresponding reduction in FVC of 0.12 L and FEV_1_ of 0.13 L for obese patients with asthma compared to non-obese patients with asthma (Table 4). Of the 189 patients extracted by the PSM method, 142 were non-obese, and 47 were obese in the group of patients without asthma. On the other hand, 130 patients were non-obese, and 59 patients were obese in the group of patients with asthma. The tendency of the results for pulmonary function testing were reproduced in that FVC, FVC percent predicted, FEV_1_, and FEV_1_ percent predicted were significantly lower in obese patients with asthma than in non-obese patient with asthma (all *p* < 0.01), but these differences were not observed between obese patients without asthma and non-obese patient without asthma (Supplemental Table 2).

**Table 3:** Characteristics of patients with and without asthma associated with obesity.

	Non-obese without asthma	Obese without asthma	*p* value	Non-obese with asthma	Obese with asthma	*p* value
*n*	1570	589		134	59	
Body mass index (kg/m^2^)	21.1 ± 2.4	28.2 ± 3.0		20.9 ± 2.0	27.7 ± 2.4	
Age	63.1 ± 17.5	62.3 ± 15.5	0.01	50.2 ± 20.2	60.1 ± 16.0	< 0.01
Sex (M/F)	802/768	326/263	0.34	48/86	21/38	0.98
Smoking history (pack-year)	14.8 ± 24.9	13.6 ± 21.5	0.56	8.5 ± 19.0	9.6 ± 17.6	0.45
**Smoking status**						
Never smoker	910 (58.0%)	347 (58.9%)	0.69	93 (69.4%)	37 (62.7%)	0.36
Ex-smoker	407 (25.9%)	142 (24.1%)	0.39	24 (17.9%)	16 (27.1%)	0.15
Current smoker	253 (16.1%)	100 (17.0%)	0.63	17 (12.7%)	6 (10.2%)	0.61
**Comorbidities**						
Hypertension	599 (38.2%)	296 (50.3%)	< 0.01	22 (16.4%)	29 (49.2%)	< 0.01
Diabetes mellitus	270 (17.2%)	136 (23.1%)	< 0.01	9 (6.7%)	12 (20.3%)	< 0.01
Hyperlipidemia	298 (19.0%)	148 (25.1%)	< 0.01	14 (10.5%)	15 (25.4%)	< 0.01
Cardiovascular diseases	234 (14.9%)	113 (19.2%)	0.02	8 (6.0%)	8 (13.6%)	0.09
**Pulmonary function test**						
FVC, percent predicted (%)	96.6 ± 15.3	96.2 ± 15.0	0.68	95.9 ± 16.5	87.9 ± 17.1	< 0.01
FEV_1_, percent predicted (%)	96.0 ± 15.2	95.6 ± 15.3	0.68	87.1 ± 19.9	75.1 ± 20.7	< 0.01

FVC, forced vital capacity; FEV_1_, forced expiratory volume in 1 s; CI, confidence interval.

**Table 4:** Univariate and multivariate analyses of FVC and FEV_1_ in obese patients without asthma versus non-obese patients without asthma and obese patients with asthma versus non-obese patients with asthma.

Obese versus non-obese in patients without asthma	Univariate analysis			Obese versus non-obese in patients with asthma	Univariate analysis		
	*β*	95% CI	*p* value		*β*	95% CI	*p* value
FVC (L)	− 0.03	− 0.07–0.01	0.14	FVC (L)	0.23	0.09–0.36	< 0.01
FEV_1_ (L)	− 0.02	− 0.05–0.01	0.23	FEV_1_ (L)	0.26	0.14–0.38	< 0.01

Obese versus non-obese in patients without asthma	Multivariate analysis*			Obese versus non-obese in patients with asthma	Multivariate analysis†		
	*β*	95% CI	*p* value		*β*	95% CI	*p* value
FVC (L)	0.00	− 0.03–0.03	1.0	FVC (L)	0.12	0.02–0.22	0.02
FEV_1_ (L)	0.01	− 0.01–0.03	0.5	FEV_1_ (L)	0.13	0.05–0.22	< 0.01

FVC, forced vital capacity; FEV_1_, forced expiratory volume in 1 s; CI, confidence interval.

*FVC and FEV_1_ were individually adjusted by confounding factors including age, sex, and smoking history.

^†^FVC and FEV_1_ were individually adjusted by confounding factors including age, sex, smoking history and dose of ICS.

### Allergic comorbidities, therapies, and laboratory data in patients with asthma focusing on obesity

Allergic rhinitis was less common in obese patients with asthma than in non-obese patients with asthma (*p* = 0.02), but atopic dermatitis and sinusitis were not different between the groups. Food allergy and drug allergy were more frequently seen in obese patients with asthma than in non-obese patients with asthma (both *p* = 0.01). In terms of therapies for the treatment of asthma, low-dose ICS was used significantly less often (*p* = 0.03), and high-dose ICS tended to be used more often (*p* = 0.05) in obese patients with asthma than in non-obese patients with asthma. The ICS doses calculated by beclomethasone equivalents were not different between the groups. Other therapies, long-acting β2 adrenergic agonists (LABAs), long-acting muscarinic antagonists (LAMAs), leukotriene receptor antagonists (LTRAs), and daily use of oral corticosteroid (OCS), were not different between the groups, but molecular targeting drugs were more often used in obese patients with asthma than in non-obese patients with asthma (*p* < 0.01), even though the absolute numbers were quite low. On laboratory testing, white blood cell counts and neutrophil counts were significantly higher in obese patients with asthma than in non-obese patients with asthma (*p* = 0.01, *p* = 0.03), and the percentages of eosinophils and eosinophil counts were not different between the two groups (Table 5).

**Table 5:** Allergic comorbidities, therapies, and laboratory data in non-obese patients with asthma and obese patients with asthma.

	Non-obese with asthma	Obese with asthma	*p* value
n	134	59	
**Allergic comorbidities**			
Allergic rhinitis	31/131 (23.7%)	6/59 (10.2%)	0.02
Atopic dermatitis	9/132 (6.8%)	3/59 (5.1%)	0.64
Sinusitis	14/133 (10.5%)	3/59 (5.1%)	0.2
Food allergy	7/130 (5.4%)	10/58 (17.3%)	0.01
Drug allergy	16/130 (12.3%)	16/58 (27.6%)	0.01
**Asthma therapy**			
Low dose of ICS	25 (18.8%)	4 (7.0%)	0.03
Moderate dose of ICS	73 (54.9%)	27 (47.4%)	0.34
High dose of ICS	17 (12.8%)	14 (24.6%)	0.05
ICS dose (mg)*	439.8 ± 282.3	496.5 ± 350.0	0.3
LABA	95 (70.1%)	43 (72.9%)	0.78
LAMA	13 (9.7%)	6 (10.2%)	0.92
LTRA	37 (27.6%)	21 (35.6%)	0.27
Daily use of OCS	16 (11.9%)	7 (11.9%)	0.72
Molecular targeting drugs	1 (0.8%)	5 (8.5%)	< 0.01
**Laboratory data**			
White blood cell (/ml)	6989.1 ± 2900.7	7743.9 ± 2402.9	0.01
Eosinophil (%)	6.3 ± 7.4	4.9 ± 5.1	0.32
Eosinophil count (/ml)	525.6 ± 1251.3	396.5 ± 487.1	0.86
Neutrophil (%)	59.9 ± 13.7	59.9 ± 9.6	0.94
Neutrophil count (/ml)	4188.0 ± 2074.2	4722.8 ± 1932.5	0.03

ICS, inhaled corticosteroid; LABA, long-acting β2 adrenergic agonist; LAMA, long-acting muscarinic antagonist; LTRA, leukotriene receptor antagonist; OCS: oral corticosteroid.

*The dose was calculated by beclomethasone equivalents.

### Impact of sex differences on obesity for patients with and without asthma

Given the sex difference in obesity-induced asthma severity augmentation^13,16,24^, the impact of obesity on parameters of pulmonary function testing was examined by sex in patients with and without asthma. In patients of both sexes without asthma, FVC percent predicted and FEV_1_ percent predicted were not different between non-obese patients and obese patients. In patients with asthma, in males, FEV_1_ percent predicted, but not FVC percent predicted, was significantly lower in obese patients than in non-obese patients (*p* = 0.03). In females, FVC percent predicted and FEV_1_ percent predicted were significantly lower in obese patients than in non-obese patients (*p* = 0.01, < 0.01 respectively) (Table S3). On multivariate analysis, FEV_1_, but not FVC, was still significant after adjustment for confounding factors including age and smoking history in both sexes (male: 0.18 [0.01–0.35], *p* = 0.04, female: 0.09 [0.01–0.17], *p* = 0.03) (Table S4).

## Discussion

In the present single-center, cross-sectional study, the impact of obesity on pulmonary function was examined in Japanese adult patients with asthma. Notably, all of the patients followed by pulmonary physicians with the disease name of asthma covered by medical insurance in our institute from 2005 to 2019 were included, and 193 patients definitely diagnosed as having asthma by a pulmonary physician were analyzed; this approach likely led to decreased rates of misdiagnosis and selection bias. In addition, the data of pulmonary function testing for patients without asthma were used for the comparison, which facilitated the evaluation of the effect of obesity in patients with asthma along with those without asthma. The present results showed that pulmonary functions were lower in patients with asthma than in those without asthma. Furthermore, FEV_1_ and FVC was negatively correlated with BMI in patients with asthma, though the correlation coefficients were relatively low, and obese patients with asthma showed significantly lower pulmonary functions than non-obese patients with asthma. Importantly, these differences were not seen between obese patients without asthma and non-obese patients without asthma.

Pulmonary function testing is an important examination for patients with asthma^6^ because decreased pulmonary function, including FEV_1_ and FVC, is associated with the severity of asthma. For example, decreased FEV_1_ is a major characteristic of severe asthma, along with increased symptoms and exacerbations^25,26^. Reduced FVC is also correlated with uncontrolled asthma defined by emergency department visits^27^ and is significantly lower in asthma patients with severe airflow obstruction than in those with moderate airflow obstruction based on their baseline FEV_1_^28^. These data and the present results show that decreased pulmonary function is an important parameter reflecting asthma severity.

There is increasing evidence that obesity is related to the severity of asthma. In Japan, To et al. examined the impact of obesity defined by a BMI greater than 25 kg/m^2^ in 492 patients with severe asthma, and they found that obesity was associated with severe acute exacerbations in females^16^. We also reported that the annual exacerbation rate was significantly higher in overweight patients than in non-overweight patients, although there was no significant difference in pulmonary function given the small sample size^17^. In a cohort of 28,016 patients with asthma in the USA, seasonal exacerbations were significantly increased in overweight patients, defined as those whose BMI was 25–29.9 kg/m^2^, and obese patients, defined as those whose BMI was greater than 30 kg/m^2^, than in those with normal BMIs^29^. Importantly, in patients with asthma, exacerbations contribute to excess lung function decline^30,31^. Therefore, the present results indicating that obesity is related to lower pulmonary function in patients with asthma are consistent with those findings.

The present results showed that obese patients with asthma had significantly worse pulmonary functions than non-obese patients with asthma (Table 3). A population-based cohort study in The Netherlands of the epidemiology of obesity showed that, in 472 patients with asthma, obesity (BMI > 30 kg/m^2^) was associated with lower FEV_1_ and FVC than non-obesity^32^. Another longitudinal study also showed that obesity was significantly associated with decreased lung function (FVC), and the risk was higher in patients with asthma than in those without asthma^33^, which supported the present results. As for the mechanisms, decreased responses to corticosteroid therapy, which is one of the factors related to the severity of asthma with obesity^21,34^, might be involved. Indeed, the present study showed that obese patients with asthma were more often treated by high-dose ICS than non-obese asthma patients, even though their pulmonary functions were low (Table 5). In addition, the previous Japanese study mentioned above showed that obesity does not affect pulmonary functions when focusing on severe asthma patients treated by high-dose ICS compared to those without obesity, in contrast to the present study which included mild to severe patients with asthma. Notably, the present blood test results showed that obese patients with asthma had higher blood neutrophils, but not eosinophils, which might be related to the effectiveness of corticosteroid therapy^35,36^. These data remind us of the possibility that obese patients with asthma might generally include more patients with resistance to corticosteroid therapy and, consequently, have reduced pulmonary function compared to non-obese patients with asthma.

Pulmonary function, especially FEV_1_, was significantly lower in obese patients with asthma than in non-obese patients with asthma, and these differences were not seen between obese patients without asthma and non-obese patients without asthma (Tables 3, 4). Furthermore, the obesity-induced decline of FEV_1_ was greater than that of FVC on correlation analysis and multivariate analysis (Fig. 3a–d, Table 4). As for the mechanisms, augmented airway and systemic inflammation induced by obesity should be considered^17,19,20,37^, but airway dysanapsis, a physiological incongruence between the growth of lung parenchyma and the caliber of the airway, might be involved in obese patients with asthma^38^. Several studies reported that airway dysanapsis, explained by high FVC, normal FEV_1_, and low FEV_1_/FVC, was more frequently seen in overweight/obese asthmatic children than in those of normal weight^39,40^. Because obesity is associated with the incidence of asthma itself^22^, the result that obesity decreased FEV_1_ featured by airway dysanapsis, considered a diagnostic factor of asthma, might be consistent.

Interestingly, the present results showed that FVC was also lower in obese patients with asthma than in non-obese patients with asthma, and these differences were not seen between obese patients without asthma and non-obese patients without asthma (Table 3). These results cannot be explained by the obesity-induced physical effects caused by mobility regulation of the diaphragm and thorax by increased fat^22,23^. While we do not yet know the reason, obesity appears to have a specific impact on FVC along with FEV_1_ in patients with asthma, but not in those without asthma. Sex differences are normally involved in the pathophysiology of asthma with obesity, and studies in Japan of asthma incidence and exacerbation related to obesity showed more impact in female than male patients^16,41^. In the present study, there were no sex differences in pulmonary function associated with obesity (Tables S3, S4).

The present study has several limitations. First, the background characteristics were different between patients with and without asthma. This might have affected the impact of obesity on the results of pulmonary function testing in patients with and without asthma. However, the results of the PSM group comparison also confirmed that pulmonary functions were lower in patients with than in those without asthma. In addition, FEV_1_ and FVC were significantly lower in obese patients with asthma than in non-obese patients with asthma. These obesity-associated differences were not observed in patients without asthma, which we believe reduced the biases, and were confirmatory. Second, patients with chronic obstructive pulmonary disease may not have been completely excluded from the patients with and without asthma, which would have affected the results of pulmonary function testing. Third, it is unclear that the observed obesity-induced decreased pulmonary function in asthma contributes to poor outcomes, especially mortality. This was not assessed, and recently, overweight was found to be associated with improvement of long-term survival in a Japanese cohort^42^. Fourth, information on waist circumference was missing, which might have affected the present results, because the distribution of abdominal and thoracic fat mass is important for obesity-induced pathophysiology^43,44^ . Fifth, the range of time differences between the day of performing pulmonary function testing and obtaining clinical information was large up to 259 days, which might affect to the present results. Sixth, pulmonary function testing was not precisely performed according to the American Thoracic Society/European Respiratory Society guideline^45^, which might also affect to the present results. Finally, the present study involved a small number of patients at a single hospital with limited ethnic diversity. To confirm the validity of the present results, multicenter, prospective studies designed with appropriate controls and larger numbers of patients should be performed.

## Conclusion

The present cross-sectional study showed that parameters of pulmonary function testing including FEV_1_ and FVC were significantly lower in obese patients with asthma than in non-obese patients with asthma. These differences were not observed between obese patients without asthma and non-obese patients without asthma. High-dose inhaled corticosteroid therapy was more common in obese patients with asthma than in non-obese patients, which might indicate that obesity-related corticosteroid resistance is the mechanism.

## Methods

### Patients and diagnosis

The purpose of the study was to clarify the effects of obesity, defined as a BMI greater than 25 kg/m^2^, on pulmonary function in patents with asthma and in those without asthma. To identify patients with asthma who underwent pulmonary function testing, 716 adult patients (age ≥ 18 years) who were followed by pulmonary physicians at Saga University Hospital with the International Classification of Diseases, 10th Revision, Clinical Modification [ICD-10-CM] code of asthma (J459) from January 2005 to March 2019 were individually reviewed by a pulmonary physician. A total of 523 patients were excluded because of lack of medical information, other pulmonary diseases including COPD, chronic cough, and bronchitis, and 193 patients with asthma diagnosed by pulmonary physicians with reference to the Global Initiative for Asthma (GINA) guidelines^46^ were identified (Fig. 1). On the other hand, to identify patients without asthma who underwent pulmonary function testing, 2710 adult patients (age ≥ 18 years) who underwent pulmonary function testing at Saga University Hospital for preoperative examination in 2019 were individually reviewed by a pulmonary physician. A total of 551 patients were excluded because of lack of medical information or complications of asthma and other diseases that contribute to decreased pulmonary function, such as chronic obstructive pulmonary disease (COPD), interstitial pneumonia, pleural effusion, and neuromuscular diseases in the medical record. Patients with a history of lung resection were also excluded, and 2,159 patients were identified as patients without asthma (Fig. 2). The surgical procedures that the 2,159 patients underwent were as follows: abdominal surgery, 447 (20.7%) patients; cervical surgery, 394 (18.2%) patients; orthopedic surgery, 294 (13.6%) patients; urological surgery, 280 (13.0%) patients; gynecological surgery, 197 (9.1%) patients; cardiac surgery, 178 (8.2%) patients; brain surgery, 127 (5.9%) patients; skin surgery, 111 (5.1%) patients; lung surgery, 76 (3.5%) patients; ophthalmic surgery, 43 (2.0%) patients; surgical construction of an arteriovenous fistula for hemodialysis, 10 (0.5%) patients; and bone marrow transplantation, 2 (0.1%) patients. An obese patient was defined as one whose BMI was > 25 kg/m^2^, referring to criteria in Japan^47^.

### Ethics approval and consent to participate

The present study was approved by the ethics committees of Saga University Hospital (approval number: 2021-11-R-01) and was performed in accordance with the 1964 Declaration of Helsinki. Informed consent of the participants was obtained in the form of opt-out on the website of Saga University Hospital, and no patients declined participation in the present study.

### Data collection

The information for smoking history, comorbidities, asthma-related therapy, and laboratory data were collected from patients’ medical records at the time point nearest to when pulmonary function testing was performed. The average time between the day of performing pulmonary function testing and obtaining clinical information was 14.0 days (range 0–259 days). The data for BMI and age were obtained when pulmonary function testing was performed. Comorbidities and allergic comorbidities were diagnosed by the physicians. Cardiovascular disease included coronary artery disease, valvular disease, cardiac arrhythmias such as atrial fibrillation, and chronic heart failure diagnosed by echocardiography. Treatments for asthma were also selected at the physicians’ discretion, and doses of inhaled corticosteroid (ICS) were divided into 3 levels, low, moderate, and high, referring to the GINA guidelines^46^. In patients with asthma, spirometry parameters without using short-acting bronchodilators and withholding of controllers such as ICS was measured in a stable condition without exacerbation and airway infection. Pulmonary function testing was performed 2 or 3 times, and the highest parameters were obtained at a single point. The FVC, percent predicted, and FEV_1_, percent predicted were calculated with the LMS method, referring to the recommendations of the Japanese Respiratory Society^48^.

### Statistical analysis

Quantitative data are expressed as means ± standard deviation (SD). The clinical data were analyzed by the Mann–Whitney U test for continuous variables and the chi-squared test for categorical variables. For correlation analysis, Pearson’s correlation coefficient was calculated to determine whether it was zero. Multivariate analysis with linear regression analysis for continuous variables and logistic regression analysis for categorical variables were performed, and the regression coefficients (*β*) were calculated. Comparing patients with and without asthma, FVC and FEV_1_ were individually adjusted by confounding factors including height, age, sex, and smoking history. Comparing non-obese patients with asthma and obese patients with asthma, FVC and FEV_1_ were individually adjusted by confounding factors including age, sex, smoking history, and ICS dose. Comparing non-obese patients without asthma and obese patients without asthma, FVC and FEV_1_ were individually adjusted by confounding factors including age, sex, and smoking history. To mitigate the risk of confounding due to differences between patients without asthma and those with asthma, the PSM method was used to balance the groups with respect to known confounders including BMI, age, sex, and smoking history. Subjects were matched 1:1 using the fitted value on the logit scale and matching using the nearest neighbor approach without replacement with a caliper of 0.05. Therefore, 189 patients from each group were extracted. Significance was considered at a p value less than 0.05. Statistical analysis was performed with JMP Pro version 14.2.0 software (SAS Institute Inc., Cary, NC, USA).

## Supplementary Information

Supplementary Information.

## Data Availability

The datasets used and analyzed during the current study are available from the corresponding author on reasonable request.
