# Supplementary material for: Obesity affects pulmonary function in Japanese adult patients with asthma, but not those without asthma
Source: Sci Rep. 2022 Sep 30;12:16457. doi: 10.1038/s41598-022-20924-y (PMC9525285; doi:10.1038/s41598-022-20924-y)
Supplement: Supplementary file 1 — Supplementary Information. [file 41598_2022_20924_MOESM1_ESM.docx]

**Supplementary Information**

**Title**

Obesity affects pulmonary function in Japanese adult patients with asthma, but not those without asthma

**Authors**

Hiroki Tashiro^1^, Koichiro Takahashi^1^, Yuki Kurihara^1^, Hironori Sadamatsu^1^, Yuki Kuwahara^1^, Ryo Tajiri^2^, Shinya Kimura^1^, Naoko Sueoka-Aragane^1^

**Affiliations**

1. Division of Hematology, Respiratory Medicine and Oncology, Department of Internal Medicine, Faculty of Medicine, Saga University, Saga, Japan

2. Clinical Research Center, Saga University Hospital, Saga, Japan

Table of contents

Table S1. Characteristics of patients with and without asthma who were extracted by the propensity score-matching method

Table S2. Characteristics of patients with and without asthma associated with obesity who were extracted by the propensity score-matching method

Table S3. Obesity-associated parameters of pulmonary function testing in patients without asthma and those with asthma by sex

Table S4. Multivariate analysis of FVC and FEV_1_ in obese patients without asthma versus non-obese patients without asthma and obese patients with asthma versus non-obese patients with asthma by sex

Table S1. Characteristics of patients with and without asthma who were extracted by the propensity score-matching method

|  | Patients without asthma | Patients with asthma | p value |
| --- | --- | --- | --- |
| n | 189 | 189 |  |
| Body mass index (kg/m^2^) | 23.1 ± 4.3 | 23.0 ± 3.8 | 0.89 |
| Haight (cm) | 158.5 ± 9.3 | 158.7 ± 9.1 | 0.47 |
| Age | 53.4 ± 18.6 | 53.0 ± 19.6 | 0.91 |
| Sex (M/F) | 65/124 | 68/121 | 0.75 |
| Smoking history (pack-year) | 7.8 ± 16.2 | 8.9 ± 18.5 | 0.54 |
|  |  |  |  |
| Comorbidities |  |  |  |
| Hypertension | 62 (32.8%) | 50 (26.5%) | 0.18 |
| Diabetes mellitus | 19 (10.1%) | 20 (10.6%) | 0.87 |
| Hyperlipidemia | 37 (19.6%) | 29 (15.3%) | 0.28 |
| Cardiovascular diseases | 17 (9.0%) | 16 (8.5%) | 0.86 |
|  |  |  |  |
| Pulmonary function test |  |  |  |
| FVC (L) | 3.07 ± 0.81 | 2.98 ± 0.91 | 0.28 |
| FVC, percent predicted (%) | 96.9 ± 13.7 | 93.2 ± 17.1 | 0.03 |
| FEV_1_ (L) | 2.49 ± 0.7 | 2.19 ± 0.81 | <0.01 |
| FEV_1_, percent predicted (%) | 96.5 ± 14.2 | 83.4 ± 21.0 | <0.01 |

Abbreviations:　FVC: forced vital capacity, FEV_1_: forced expiratory volume in 1 second

Table S2. Characteristics of patients with and without asthma associated with obesity who were extracted by the propensity score-matching method

|  | Non-obese without asthma | Obese without asthma | p value |  | Non-obese with asthma | Obese with asthma | p value |
| --- | --- | --- | --- | --- | --- | --- | --- |
| n | 142 | 47 |  |  | 130 | 59 |  |
| Body mass index (kg/m^2^) | 21.2 ± 2.3 | 28.9 ± 3.7 |  |  | 20.9 ± 2.0 | 27.7 ± 2.4 |  |
| Age | 53.3 ± 19.3 | 53.7 ± 16.6 | 0.85 |  | 49.8 ± 20.3 | 60.1 ± 16.0 | <0.01 |
| Sex (M/F) | 48/94 | 17/30 | 0.77 |  | 47/83 | 21/38 | 0.94 |
| Smoking history (pack-year) | 7.3 ± 15.6 | 9.5 ± 17.8 | 0.8 |  | 8.5 ±19.0 | 9.6 ± 17.6 | 0.45 |
|  |  |  |  |  |  |  |  |
| Comorbidities |  |  |  |  |  |  |  |
| Hypertension | 42 (29.6%) | 20 (42.6%) | 0.11 |  | 21 (16.2%) | 29 (49.2%) | <0.01 |
| Diabetes mellitus | 9 (6.3%) | 10 (21.3%) | <0.01 |  | 8 (6.2%) | 12 (20.3%) | <0.01 |
| Hyperlipidemia | 23 (16.2%) | 14 (29.8%) | 0.05 |  | 14 (10.8%) | 15 (25.4%) | 0.01 |
| Cardiovascular diseases | 8 (5.6%) | 9 (19.2%) | <0.01 |  | 8 (6.2%) | 8 (13.6%) | 0.1 |
|  |  |  |  |  |  |  |  |
| Pulmonary function test |  |  |  |  |  |  |  |
| FVC (L) | 3.04 ± 0.80 | 3.14 ± 0.84 | 0.65 |  | 3.12 ± 0.91 | 2.67 ± 0.84 | <0.01 |
| FVC, percent predicted (%) | 95.9 ± 15.0 | 98.2 ± 11.6 | 0.42 |  | 95.6 ± 16.6 | 87.9 ± 17.1 | <0.01 |
| FEV_1_ (L) | 2.47 ± 0.7 | 2.54 ± 0.72 | 0.72 |  | 2.36 ± 0.8 | 1.82 ± 0.7 | <0.01 |
| FEV_1_, percent predicted (%) | 96.4 ± 14.0 | 98.6 ± 12.7 | 0.43 |  | 87.3 ± 20.0 | 75.1 ± 20.7 | <0.01 |

Abbreviations:　FVC: forced vital capacity, FEV_1_: forced expiratory volume in 1 second

Table S3. Obesity-associated parameters of pulmonary function testing in patients without asthma and those with asthma by sex

| Male | Non-obese without asthma | Obese without asthma | p value |  | Non-obese with asthma | Obese with asthma | p value |
| --- | --- | --- | --- | --- | --- | --- | --- |
| n | 802 | 326 |  |  | 48 | 21 |  |
| Pulmonary function test |  |  |  |  |  |  |  |
| FVC, percent predicted (%) | 95.8 ± 14.9 | 95.6 ± 14.8 | 0.65 |  | 95.8 ± 19.5 | 87.6 ± 16.8 | 0.16 |
| FEV_1_, percent predicted (%) | 95.1 ± 15.3 | 94.2 ± 15.3 | 0.42 |  | 81.5 ± 19.5 | 69.1 ± 21.3 | 0.03 |
|  |  |  |  |  |  |  |  |
| Female | Non-obese without asthma | Obese without asthma | p value |  | Non-obese with asthma | Obese with asthma | p value |
| n | 768 | 263 |  |  | 86 | 38 |  |
| Pulmonary function test |  |  |  |  |  |  |  |
| FVC, percent predicted (%) | 97.3 ± 15.7 | 97.0 ± 15.1 | 0.98 |  | 96.0 ± 14.6 | 88.1 ± 17.6 | 0.01 |
| FEV_1_, percent predicted (%) | 97.0 ± 15.0 | 97.3 ± 15.2 | 0.65 |  | 90.3 ± 19.5 | 78.4 ± 19.9 | <0.01 |

Abbreviations:　FVC: forced vital capacity, FEV_1_: forced expiratory volume in 1 second

Table S4. Multivariate analysis of FVC and FEV_1_ in obese patients without asthma versus non-obese patients without asthma and obese patients with asthma versus non-obese patients with asthma by sex

| Male patients without asthma | | | |  | Male patients with asthma | | | |
| --- | --- | --- | --- | --- | --- | --- | --- | --- |
| obese versus non-obese | Multivariate analysis* | | |  | obese versus non-obese | Multivariate analysis* | | |
|  | β | 95% CI | p value |  |  | β | 95% CI | p value |
| FVC (L) | 0.00 | -0.04 – 0.04 | 0.85 |  | FVC (L) | 0.16 | -0.06 – 0.39 | 0.16 |
| FEV_1_ (L) | 0.01 | -0.02 – 0.05 | 0.37 |  | FEV_1_ (L) | 0.18 | 0.01 – 0.35 | 0.04 |
|  |  |  |  |  |  |  |  |  |
| Female patients without asthma | | | |  | Female patients with asthma | | | |
| obese versus non-obese | Multivariate analysis* | | |  | obese versus non-obese | Multivariate analysis* | | |
|  | β | 95% CI | p value |  |  | β | 95% CI | p value |
| FVC (L) | 0.01 | -0.02 – 0.04 | 0.56 |  | FVC (L) | 0.08 | -0.01 – 0.17 | 0.09 |
| FEV_1_ (L) | 0.00 | -0.02 – 0.03 | 0.72 |  | FEV_1_ (L) | 0.09 | 0.01 – 0.17 | 0.03 |

Abbreviations:　FVC: forced vital capacity, FEV_1_: forced expiratory volume in 1 second, CI: confidential interval

*FVC and FEV_1_ were individually adjusted by confounding factors including age and smoking history.
